# Worldwide Prevalence of *mcr*-mediated Colistin-Resistance *Escherichia coli* in Isolates of Clinical Samples, Healthy Humans, and Livestock—A Systematic Review and Meta-Analysis

**DOI:** 10.3390/pathogens11060659

**Published:** 2022-06-08

**Authors:** Carlos Bastidas-Caldes, Jacobus H. de Waard, María Soledad Salgado, María José Villacís, Marco Coral-Almeida, Yoshimasa Yamamoto, Manuel Calvopiña

**Affiliations:** 1 One Health Research Group, Faculty of Engineering and Applied Sciences, Biotechnology Section, Universidad de Las Américas, Quito 170124, Ecuador; maria.salgado.garce@udla.edu.ec (M.S.S.); maria.villacis.barahona@udla.edu.ec (M.J.V.); 2 Programa de Doctorado en Salud Pública y Animal, Facultad de Veterinaria, Universidad de Extremadura, 06006 Mérida, Spain; 3 One Health Research Group, Faculty of Health Sciences, Universidad de Las Américas, Quito 170124, Ecuador; jacobus.dewaard@udla.edu.ec (J.H.d.W.); manuel.calvopina@udla.edu.ec (M.C.); 4 The United Graduate School of Drug Discovery and Medical Information Sciences, Gifu University, Gifu 501-1193, Japan; yyamamot@gifu-u.ac.jp

**Keywords:** colistin, resistance, *mcr*, *Escherichia coli*, clinical, community, animals

## Abstract

**Background:** Antimicrobial resistance is a serious public-health problem throughout the world. *Escherichia coli*, the most common Gram-negative microorganism, has developed different resistance mechanisms, making treating infections difficult. Colistin is considered a last-resort drug in the treatment of infections caused by *E. coli*. Plasmid-mediated mobile-colistin-resistant (*mcr*) genes in *E. coli*, now disseminated globally, are considered a major public-health threat. Humans, chickens, and pigs are the main reservoirs for *E. coli* and the sources of antibiotic resistance. Hence, an up-to-date and precise estimate of the global prevalence of *mcr* resistance genes in these reservoirs is necessary to understand more precisely the worldwide spread and to more effectively implement control and prevention strategies. **Methodology**: Publications were identified in the PubMed database on the basis of the PRISMA guidelines. English full-text articles were selected from December 2014 to March 2021. Descriptive statistics and a meta-analysis were performed in Excel and R software, respectively. Colistin resistance was defined as the molecular-genetic detection of the *mcr* genes. The crude and estimated prevalence were calculated for each host and continent. The studies were divided into two groups; community-based when they involved isolates from healthy humans, chickens, or pigs, and clinical studies when they involved only hospital, outpatient, or laboratory isolates. **Results**: A total of 1278 studies were identified and 218 were included in this systematic review and meta-analysis, divided into community studies (159 studies) and clinical studies (59 studies). The general prevalence of *mcr*-mediated colistin-resistant *E. coli* (**mcrMCRE**) was 6.51% (n = 11,583/177,720), reported in 54 countries and on five continents; Asia with 119 studies followed by Europe with 61 studies registered the most articles. Asia reported the major diversity of *mcr*-variants (eight of nine, except *mcr-2*). Worldwide, chickens and pigs proved to be the principal reservoir of *mcr* with an estimated prevalence of 15.8% and 14.9%, respectively. Healthy humans and clinical isolates showed a lower prevalence with 7.4% and 4.2% respectively. **Conclusions**: In this systematic review and meta-analysis, the worldwide prevalence of *mcr* in *E. coli* isolated from healthy humans, chickens, and pigs was investigated. A wide prevalence and distribution of *mcr* genes was demonstrated on all continents in *E. coli* isolates from the selected reservoirs. Understanding the epidemiology and occurrence in the reservoirs of *mcr* in *E. coli* on different continents of the world facilitates tracing how *mcr* genes are transmitted and determining the infection risks for humans. This knowledge can be used to reduce the incidence of zoonotic transmission by implementing the appropriate control programs.

## 1. Introduction

The emergence of antibiotic-resistant bacteria is one of the most urgent human and veterinary health problems worldwide, representing a major threat to food security [1,2,3]. Serious infections caused by bacteria exhibiting multiple resistances to most commercially available antibiotics such as fluoroquinolones, aminoglycosides, carbapenem, and ß-lactams have been increasing in recent years. The lack of development of new antimicrobial agents has led to the reevaluation of colistin [4,5,6] with the drug now regaining clinical value as a last-resort pharmacon in the treatment of infections caused by Gram-negative bacteria [7,8]. Over the last few years, colistin was used excessively in veterinary medicine as a prophylactic treatment and as a livestock-growth promoter, resulting in the consequent appearance of new resistance mechanisms in bacteria [9,10]. 

*Escherichia coli* is a bacterium commonly found in the gut of humans and warm-blooded animals. Most strains of *E. coli* are harmless. Certain strains, however, can cause severe foodborne disease. For example, a systematic review revealed a substantial burden of *E. coli* bacteremia in high income countries, with an estimated incidence of 48 cases per 100,000 person-years [11]. With respect to antibiotic susceptibility, a study in the USA with 67,583 patients with invasive *E. coli* infections found that 9.18% were resistant to extended-spectrum cephalosporins, 28.2% to fluoroquinolones, and 0.14% to carbapenems. Resistance to extended-spectrum cephalosporins increased from 5.46% to 13.0% during the 8-year study period [12].

The transmission of *E. coli* between animals and humans is primarily through the consumption of contaminated food such as raw or undercooked ground meat products and raw milk or the contamination of water with animal feces. Contaminated surfaces and kitchen utensils can also lead to infection. This microorganism furthermore has a great capacity to accumulate antibiotic-resistance mechanisms mainly through horizontal gene transfer [13]. Among the acquired mechanisms are the genes that encode carbapenemases, ß-lactamases, methylases, and quinolone resistance; while in recent years the mobile-colistin-resistance (*mcr*) gene responsible for insensitivity to that antibiotic has become prevalent. Mobile genetic elements—such as transposons, multiple resistance plasmids, and gene cassettes in integrons—play a key role in the dissemination of these resistance genes, causing treatment failure in human and veterinary medicine [14,15,16]. 

With respect to colistin resistance, chromosomal resistance was initially the principal mechanism, where structural modifications of the bacterial lipopolysaccharide were the reason for that resistance in Gram-negative bacteria. Several systems like PhoPQ and PmrAB or mgrB exhibited phenotypic changes caused by mutations. As a result, the effective binding between the lipopolysaccharide present in the bacterial membrane and colistin was prevented [17]. In 2015, however, a new resistance mechanism was described; the *mcr* gene. This gene encodes a transferase enzyme that catalyzes a modification of the lipid-A lipopolysaccharide of the bacterium [18]. Being a transposable genetic element, *mcr* was found within different bacterial plasmids such as the IncX4 and IncHI2, hence facilitating a horizontal gene transfer [14]. Since that time, studies of Enterobacteria harboring *mcr* genes have been reported worldwide and to date variants ranging from *mcr-1* to *mcr-10* have been described [18,19,20,21,22,23,24,25,26]. Bacteria that carry these genes have been isolated from poultry, pigs, cattle, and food products derived from those animals, but also in human clinical isolates. Notwithstanding, few studies have carried out an in-depth, comprehensive analysis within the one-health context, i.e., one linking the health of humans closely connected to that of the animals within the same shared environment [27,28,29]. 

Currently, little is still known about the impact of the rapid spread of the *mcr* over the entire world’s continents. The World Health Organization recommends an enhancement in the detection and epidemiologic surveillance of microorganisms possessing this resistance, with one of the control strategies being the disclosure of worldwide information concerning colistin resistance in order to alert healthcare workers and decision-making entities [30,31,32,33]. Therefore, the present systematic review and meta-analysis aims at compiling the available information about *mcr* in *E. coli* and estimating the prevalence in three of the main hosts (pigs, chickens, and humans) along with the gene’s distribution over the different continents of the world.

## 2. Results

### 2.1. Studies Selection 

**Figure 1:**
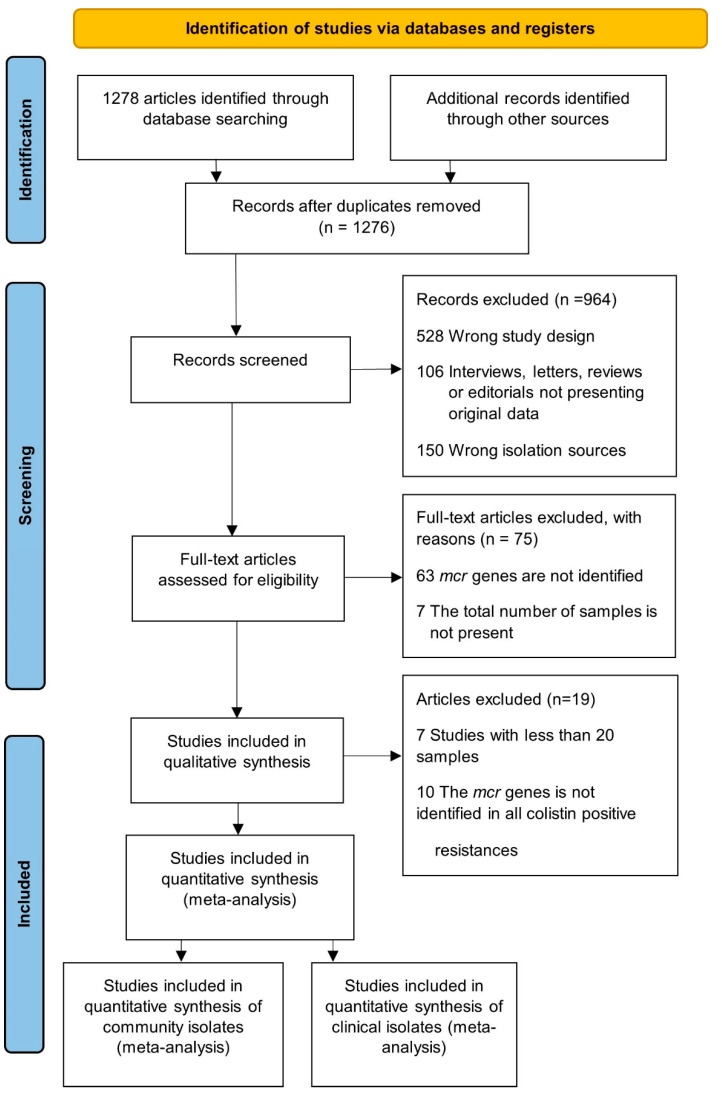
PRISMA flow diagram of the review process; selection and inclusion of studies for community and clinical data. Flow diagram for the review process involving the selection and inclusion of studies for community and clinical data from Preferred Reporting Items for Systematic Reviews and Meta-Analyses.

From an initial number of 1278 articles, 218 studies were included for quantitative synthesis. Of the studies, 72.9% (n = 159/218) corresponded to community studies that involved healthy humans, chickens and/or pigs. The other 27.1% (n = 59/218) corresponded to clinical studies where the samples were obtained from patients with an infectious disease (Figure 1) (Supplementary Material Tables S1–S4).

### 2.2. Global Spread of mcr in Escherichia coli 

**Figure 2:**
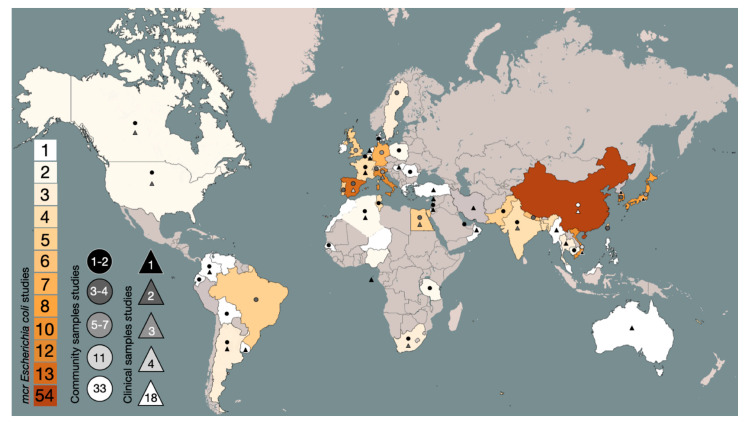
Global spread of *mcr* genes in *E. coli.* The countries colored from red-orange to yellow plus white represent the total number of studies for those countries according to the key to the lower left, while the countries in gray are without studies. The triangles in the black-to-white scale represent the number of studies with community samples (healthy humans, pigs, and/or chickens). The circles in the black-to-white scale represent the number of studies with clinical samples.

Five continents reported data, with the 218 publications accounting for a total of 54 countries that reported mcrMCRE. The 119 studies on the continent of Asia were distributed over 19 countries, followed by Europe with 61 studies distributed over 16 countries, Africa with 20 studies in nine countries, the Americas (North, Central, and South) with 17 studies in nine countries, and Oceania with one study in Australia (Figure 2).

We found China to be the country with the most frequently reported incidence of *mcr* with 54 studies—followed by Spain with 13 studies; Italy with 12; South Korea and Vietnam with 10; Japan with eight; Germany with seven; Nepal with six; Brazil, Pakistan, Taiwan, United Kingdom, Egypt, and Portugal with five; France, India, Bangladesh, and Tunisia with four; Argentina, Belgium, Sweden, Switzerland, Thailand, and South Africa with three; and the United States, Cambodia, Lebanon, Nigeria, Poland, Tanzania, Algeria, Canada, and Qatar with two. Finally, several countries reported a single study such as Australia, Bolivia, Denmark, Ecuador, Ireland, Romania, Senegal, Venezuela, Myanmar, Colombia, Hungary, Greece, Israel, Oman, Morocco, Philippines, Malaysia, Netherlands, Turkey, Uruguay, Sao Tomé, and Prince. Figure 2 details the distribution between the clinical and community studies in each country.

In all the studies, nine variants of *mcr* genes were reported: *mcr-1* through *mcr-9*. The distribution of the variants over the world map was highly diverse, with Asia exhibiting the greatest diversity in the prevalence of all nine. On the European continent, the variants *mcr-1* through *mcr-5* were reported, while the Americas documented only the *mcr-1* and *mcr-3*. Finally, Africa registered the *mcr-1*, *mcr-5*, and *mcr-8* variants, but Oceania only the *mcr-1*.

The worldwide distribution of *mcr* in *E. coli* (Table 1) manifested a wide variation. The total crude prevalence was 6.52% (n = 11,583/177,720) at a prevalence of 8.71% (n = 15,001/172,140) in non-clinical isolates and one of 1.76% (n = 1020/58,033) in clinical isolates. Africa was the continent with the highest gross prevalence of *E. coli* antibiotic resistance mediated by *mcr* genes at 10.1% (n = 273/2715), followed closely by Asia at 9.24% (n = 8381/90,707). The Americas at 7.65% (n = 624/8161) and Europe at 3.03% (n = 2305/76,137) exhibited the lowest prevalence of *mcr* genes.

The meta-analysis by continents indicated a heterogeneity among the studies. Asia with a total of 119 studies reported an estimated prevalence of 11.5% (8.9–14.7) at a 95% CI and an I^2^ = 99% (Supplementary Material Figure S1) and Europe with 61 studies one of 9.1% (5.7–14.1) at a 95% CI and an I^2^ = 99% (Supplementary Material Figure S2). The 20 studies in Africa registered a prevalence of 16.7% (8.3–30.9) at a 95% CI and an I^2^ = 95% (Supplementary Material Figure S3), and finally, the 17 studies in the Americas indicated an estimated prevalence of 21.4% (7.7–46.9) at a 95% CI and an I^2^ = 98% (Supplementary Material Figure S4).

### 2.3. The mcr in Healthy Humans, Chickens, and Pigs

Community studies evidenced a wide distribution of host and samples. The most commonly studied hosts worldwide were chickens with 94 studies with *mcr* in *E. coli* indicating a gross prevalence of 10.4% (n = 7134/68,362), followed by 85 studies on pigs at 8.80% (n = 7089/80,600) and 30 studies on healthy humans at 3.35% (n = 789/23,585). The samples of the studies originated from one, two, or three host sources, with 52 being carried out with samples from chickens, 48 from pigs, and 15 only from healthy humans. Other studies combined samples; with seven studies using samples from chickens and humans, two samples from pigs and humans, 29 samples from pigs and chickens, and six samples from all thre3 hosts (humans, chickens, and pigs).

The hosts registered in the meta-analysis of community studies proved to be heterogeneous for *mcr* in *E. coli* isolates. The meta-analysis of the studies on chickens registered the highest estimated prevalence of 15.8% [11.7–20.9] at a 95% CI and an I^2^ = 98% (Supplementary Material Figure S5), followed by those on pigs at an estimated prevalence of 14.9% (10.8–20.1) at a 95% CI and an I^2^ = 99% (Supplementary Material Figure S6). Finally, healthy humans exhibited an estimated prevalence of 7.4% (3.9–13.6) at a 95% CI and an I^2^ = 98% (Supplementary Material Figure S7). Table 1 summarizes the comparisons between the gross and estimated prevalence.

**Figure 3:**
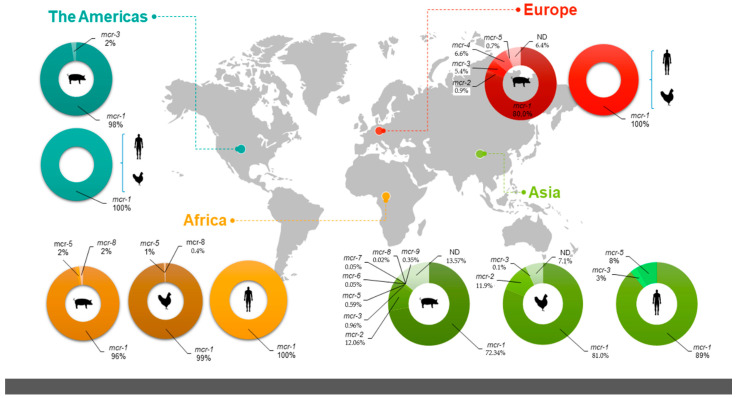
Global distribution of prevalence and diversity of *mcr* variants of *E. coli* isolates described in community studies of healthy humans, pigs, and chickens. The colored pie charts represent the percent distribution of *mcr* variants in each continent—light green, Asia; red, Europe; yellow, Africa; dark green, the Americas. The small figure silhouettes indicate the hosts (healthy humans, pigs, or chickens).

The *mcr-1* variant is predominant and widely distributed in all the continents and hosts. The studies documented that the diversity and abundance of the variants of *mcr* genes found in animals and healthy humans was greater in Asia, with eight variants (*mcr-1*–*mcr-3*, *mcr-5*–*mcr-9*);,followed by Europe with five (*mcr-1*–*mcr-5*), Africa with three (*mcr-1*, *mcr-5*, and *mcr-8*), and finally the Americas with only two (*mcr-1* and *mcr-3*). Pigs presented a greater diversity of *mcr* variants than the other hosts, reaching up to eight in Asia and five in Europe. Chickens and humans have one to three variants depending on their geographic location (Figure 3).

### 2.4. mcr in Clinical Samples

The 59 clinical studies comprising 58,033 samples of clinical or ambulatory origin manifested less diversity of *mcr*-gene variants, with only four variants being observed worldwide (*mcr-1*–*mcr-3*, and *mcr-5*). Because of the variability of the origin of the clinical samples, the latter were grouped into six categories: blood, feces, urine, respiratory, body fluids, and other samples (Supplementary Material Table S5). The continent that displayed the most diversity in their clinical studies was Asia with thre3 variants (*mcr-1*–*mcr3*), followed by Europe with two variants (*mcr-1* and *mcr-2*), the Americas with an additional two variants (*mcr-1* and *mcr-5*), and finally Africa with only one variant, the *mcr-1*.

**Figure 4:**
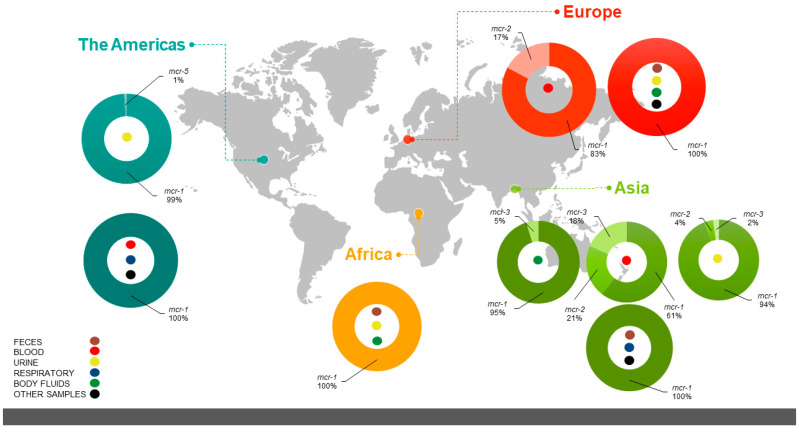
Global distribution of prevalence and diversity of *mcr* variants of *E. coli* isolates described in clinical studies. The colored pie charts represent the percent distribution of *mcr* variants in each continent—light green, Asia; red, Europe; yellow, Africa; dark green, the Americas. The colored circles indicate the nature of the clinical sample constituting the source of the isolate: brown circles, feces; red circles, blood; yellow circles, urine; blue circles, respiratory; green circles, body fluids; and black circles, other samples.

Despite the low diversity, the continent with the highest prevalence of the *mcr* gene in clinical samples was Africa at 7.58% (n = 53/699), where *mcr-1* was the only reported gene, followed by the Americas at 3.59% (n = 436/12,128) distributed between the *mcr-1* and *mcr-5* variants, with Asia and Europe reporting the lowest respective prevalence of *mcr* genes at 1.56% (n = 745/47,611) and 0.522% (n = 144/27,600). Figure 4 illustrates the distribution, frequency, and diversity of the variants in relation to the origin of the samples. Like the previous meta-analyses, those for the 59 clinical studies revealed a high heterogeneity with an estimated prevalence of 4.2% (2.4–7.3) at a 95% CI and an I^2^ = 98% (Supplementary Material Figure S8).

## 3. Discussion

### 3.1. Global Prevalence of mcr in the Hosts Studied 

To the best of our knowledge, the present meta-analysis is the first systematic review of mcrMCRE that links the resistance to colistin in nonclinical and clinical samples of humans to two principal feed animals (pigs and chickens) in a one-health context as cited above. The results of this review have enabled us to compare the estimates of the prevalence and global geographical distribution of *mcr* and its variants within the mcrMCRE in the different continents and the principal hosts. The present study revealed an increase in the dispersion of *mcr* with respect to the number of countries reporting cases and the diversity of variants over the findings from previous similar reviews covering just 31 [34] and 47 [1] countries. Asia proved to be the continent with the most studies and countries that reported *mcr*. Similarly, Europe and the Americas—in countries such as Spain, Italy, Germany, United States, Brazil, and Argentina—have reported an increase in *mcr*-mediated colistin resistance in pathogenic *E. coli*.

This rapid dissemination can be attributed to the ability of *mcr* genes to be dispersed by horizontal transfer via plasmids and transposons. Up to 11 types of plasmids capable of carrying *mcr* genes have been reported such as IncI2, IncHI2, and IncX4 [35], along with transposable elements such as ISApl1 [34]. We can assume that these genetic transmissions can be favored by the exposure to persistent antibiotic pressure at low concentrations of colistin or other polymyxins. That pressure, in turn, may be especially linked to veterinary medicine in treatments for infection or prophylaxis and/or the latter rationale in the frequent use of colistin-containing feed in chickens and pigs to prevent infection [9,36]. This practice is consistent with the high estimated prevalence in the meta-analysis for *mcr* in those respective domestic animals of 15.8% and 14.9% compared to the lowest estimated prevalence for healthy humans and clinical samples at 7.4% and 4.2%, respectively. The latter values being much lower does not, however, necessarily mean that resistance to colistin may be considered less of a threat to public health: instead, those figures may represent merely the surface of the problem. The environment and domestic animals seem to have a substantial influence on humans. Studies have indicated that touristic travels to rural areas in countries with a high or unknown prevalence could be contributing to just such an influence [21,37]. In addition, world trade, little control or prevention in the food chain, and permanent close contact with backyard animals in developing countries can increase the risk of zoonotic transmissions with *mcr* [38].

China ranks first in reporting on studies of *E. coli* containing *mcr*, because that country is considered the one with the highest consumption of colistin in agriculture, which circumstance does not apply to certain countries such as the United States, Argentina, and those in the EU that banned its use in animal production and in human treatment [39,40]. Other countries, however, with permissive laws about colistin use have also manifested a higher prevalence of *mcr* [20,41].

### 3.2. mcr Variants Worldwide

Since the first report of the *mcr-1* gene in China, 10 variants and many sub-variants have been found. Moreover, the greater dissemination of the *mcr-1* in bacterial isolates of *E. coli* may have resulted because that variant was the first one identified, with its initial emergence having been almost a decade before being isolated for the first time [42]. Indeed, retrospective studies revealed that isolates of *E. coli* had been analyzed from sick pigs within the period from 1991 to 2014 from which animals the *mcr* genes were first identified [43]. In addition, not all studies that have evaluated colistin resistance have determined the presence of all the reported variants of the *mcr* genes, where many studies only performed polymerase-chain-reaction assays with specific primers designed to detect a particular variant of the gene. That bias in the screening could well explain why the other variants of the *mcr* genes (*mcr-2*–*mcr-10*) have been less frequently reported [44,45].

Asia and Europe have more recently registered a wide diversity of *mcr* variants at eight and five, respectively, thus suggesting not only a considerable spread within those continents but also a constantly changing epidemiology. Polymyxins, mainly colistin, are one of the most widely sold antimicrobial groups worldwide [41,46]. That widespread usage is consistent with the diversity of variants founded in pigs in those two continents. This phenomenon can be explained by the continued usage of colistin as a growth promoter by certain countries in Asia and Europe [47]. In Asia, Thailand represents the highest diversity country, having six different variants of *mcr*. In Europe, Spain was found to have four variants in several studies. Italy is the country with the second highest use of polymyxins in veterinary medicine, which practice could explain why the variants of this gene are widely distributed within that region [48]. Furthermore, some significant studies in Belgium, Italy, and Spain indicated the coexistence of *mcr-1, mcr-3*, and/or *mcr-4* in isolates of *E. coli* obtained from cattle feces. These findings point to practices that could be responsible for increasing the spread and zoonotic risk in the continent [49,50,51].

### 3.3. Present and Future Implications

Different animal species have evidenced a wide distribution of *mcr* [52,53,54,55,56]. In addition to pigs and chickens, several hosts—such as cows, poultry, and domestic animals like dogs and cats—have been reported to carry the *mcr* gene in Gram-negative bacteria and then to subsequently transfer colistin resistance to humans. [57,58,59]. The high prevalence of 15.8% [11.7–20.9] and 14.9% [10.8–20.1] encountered in studies carried out in chickens and pigs, respectively, may have occurred because those animals are widely used for human consumption. This practice should promote a real interest in ensuring safety in the raising of these animals for food worldwide. Consequently, the increasing dispersion and diversity of variants encountered in this systematic review should be considered a red flag. Furthermore, many countries lack data, either due to low resources, the absence of active surveillance programs, or even a vested interest on the part of local governments in not making public such contraindicated uses of antibiotics by the livestock industry. These findings should constitute issues of major concern that will directly affect the immediate and long-term future implications for veterinary medicine and public health. [60,61].

Because little is known about the prevalence of *mcr* in the intestinal microbiota of these animals, an evaluation of the presence of these genes in the different food-production chains within the various countries is indeed urgent. Studies have been carried out on the prevalence of *mcr* in chickens and pigs that point to the livestock industry as the main source responsible for the spread and increase of colistin resistance in enterobacteria [62,63,64]. In particular, attention should be paid to commensal bacteria such as *E. coli* with easy inter-host spread and a wide broad capability of horizontal gene transfer. A retrospective study carried out in Italy in 2014 and 2015 reported isolates of colistin-resistant *E. coli* and *Salmonella* sp. with *mcr* in food-producing animals (chickens, pigs, and cattle). That study determined that the bacterial species with the greater resistance to colistin was *E. coli*, thus underscoring the probability of a horizontal transfer of *mcr* between commensal bacteria and the main pathogens transmitted by food [58].

Zoonotic pathogens involved in resistance to antimicrobials present in meat foods represent a direct danger to public health [65,66]. The one-health concept cited above recognizes that human health is integrally connected to the health of animals and the environment [67,68,69]. As stressed throughout this review, a notable prevalence of *E. coli* with *mcr* has been evidenced in consumer animals such as pigs and chickens. In contrast, the prevalence in human isolates is described as relatively low, both in community and clinical studies. These data agree with the estimated prevalence obtained by the meta-analysis where chickens and pigs worldwide were found to exhibit higher percentages of *mcr* than healthy humans and clinical cases. Of interest to us was that the estimated prevalence in all the meta-analyses carried out here was higher than the corresponding gross prevalence, which difference could point to an underestimation of the prevalence of *mcr*. These data should be considered as a matter of concern for public health in view of the easy zoonotic and environmental transmission [44,45,66,70].

Finally, this study has documented data that seek to help in decision-making to reduce mcrMCRE circulation in the environment and in animals and humans. Governments must need focus on common efforts in the use of antibiotics in animals in the face of the present resistance phenomenon and the continuous increasing demand for animal products for human consumption [71,72]. A significant effort has been made in developed countries to control and reduce the spread of *mcr*, where a ban on the free use of colistin in animals has apparently reduced the incidence of *mcr-1*-harboring IncX4-Type plasmids, whose presence is associated with a high dispersal capability in enterobacteria. Notwithstanding, this policy has not yet pervaded the global panorama: indeed, many developing countries—mainly in Africa, South America, and Asia—still manifest weak national drug-regulatory authorities, inefficient antibiotic policies, and erratic and unregulated access to antibiotics,

### 3.4. Limitations

This study has certain limitations. The first is that only studies with molecular-genetic methods for the detection of *mcr* resistance are included, with phenotypic methods being excluded because of the discrepancies and problems associated with those studies on the antibiotic colistin. The increase in Gram-negative bacteria with multiple resistances necessitates the use of an accurate method for colistin-susceptibility testing. The European Committee for Antimicrobial Susceptibility Testing (EUCAST) and the Clinical and Laboratory Standards Institute (CLSI) recommend the standard broth-micro-dilution (BMD) method for testing the minimum inhibitory concentration (MIC) of colistin as the most appropriate. This method was thus the one most frequently used in the studies. Other testing methods—such as disk diffusion, gradient diffusion, the E-test, and agar dilution—are not recommended until further investigative studies on their accuracy have been performed. [73]. The BMD method with colistin, however, was associated with methodologic problems since colistin binds to the plastic of the polystyrene trays, thus reducing the effective concentration in the broth and consequently altering the MIC values. [74,75,76,77,78]. Recently a joint EUCAST-CLSI working group decided that the recommendations of the International Standardization Organization should be met, with the tests being carried out with colistin sulfate without the addition of surfactants—where those amphipaths do not improve the performance of the assay and in fact act synergistically with colistin [79].

Gradient tests—namely, E-tests, disk diffusion, and semi-automated antimicrobial-susceptibility devices such as VITEK-2 and Thermo Scientific Sensititre—have been widely used in clinical laboratories despite advice from CLSI and EUCAST. A variety of studies comparing the use of different antimicrobial-susceptibility methods to find the most suitable one has determined that gradient tests generally underestimate the MICs of colistin, thus resulting in a significant number of false susceptibilities. These findings substantiated that diffusion tests were not reliable and accordingly led to a recommendation of the use of BMD methods [80,81]. In addition, variations in bacterial promoters are associated with the expression of *mcr-1* and consequently with the level of colistin resistance. The presence of the gene per se will not always indicate resistance so that strains harboring the *mcr* locus can nevertheless be phenotypically sensitive to colistin [82].

Another limitation is that the study does not include the coexistence of genes involved in other types of resistance. Bacteria can exhibit a multiple resistance phenotype due to the accumulation of resistance plasmids, genes encoding the resistance of a specific agent, and/or the action of efflux pumps [83]. These are the main mechanisms of *E. coli* that contain the *mcr* gene for manifesting a resistance to other antimicrobials also. The use of colistin as an antibiotic in the last line of treatment for bacteria with multiple resistances may be one of the reasons why most of the isolates that were identified as positive for *mcr* genes also displayed resistances to other antibiotics. A study carried out in Spain analyzed the co-occurrence of different variants of the *mcr-1*, *mcr-4*, and *mcr-5* genes in *E. coli* that presented multiple resistances to other drugs isolated from swine farms. The authors determined that the swine industry was a reservoir of colistin-resistant *E. coli*, which status was accompanied by other additional risk genes such as the extended-spectrum ß-lactamase (ESBL) and the cefotaxime-hydrolyzing ß-lactamase *bla*_CTXM_. Consequently, since pigs are animals for human consumption, they could be spreading a cocktail of multiple resistances [19].

The possession of a wide distribution of ESBL genes may be related to a coexistence with *mcr* genes because the latter are so widely distributed in the same food-producing animals. This extensive distribution of those genes can be attributed to an inadequate combination of multiple classes of broad-spectrum antibiotics, while the rapid increase in the occurrence of ESBL apparently also enhances the selective pressure for colistin resistance [84]. Colistin and ß-lactams can damage the cell walls of bacteria by disrupting the outer membrane and by inhibiting peptidoglycan synthesis, respectively. Maintaining the integrity of the cell membrane has become the main mechanism for the bacteria to survive the onslaught of antibiotics, which necessity can lead to a high prevalence of positive *mcr* and ESBL genes isolated. The plasmids that carry the *mcr-1* gene in most ESBL isolates are similar to the one that carries the ESBL genes, pHNSHP45. Because of the great resemblance of these resistance loci within the genetic context, the incorporation of ESBL into the bacterial genome increases the probability of the eventual acquisition of the *mcr-1* gene in isolates that do not yet possess that locus. Moreover, the study cited also mentions having detected the *mcr-1* gene and the *bla*_CTX-M-1_ gene together in the same large, conjugated IncHI2-type plasmid [85,86].

Plasmids carrying the blaNDM-1 gene also transport several other genes that confer resistance to all aminoglycosides, macrolides, and sulfamethoxazole, thus, making those isolates resistant to multiple drugs [87]. Plasmids that carry the gene for carbapenemase can carry up to 14 other determining genes for resistance to other antibiotics and transfer this resistance to other bacteria, resulting in multiple resistance phenotypes among which *mcr-1* expression has also been present. Because of this association, blaNDM genes have also possibly been identified in many of the isolates that were positive for *mcr* in the systematic review studies. A study to identify the co-transfer of those genes revealed the presence of *mcr-1* and blaNDM-5 in the same hybrid plasmid—IncX3-X4—of *E. coli* isolates, thus indicating that the possibility of finding these genes simultaneously in the same microorganism was high [85,88]. The coexistence of *mcr* and the tetracycline-resistance genes *tetA* and *tetB* may have occurred because colistin and tetracycline have been widely used in veterinary medicine and in clinical practice where the dosage and use of one alone has not been adequate. In addition, the selection pressure of the environment can likewise influence the greater spread of these genes [89].

Furthermore, we consider that another limitation of this study is the focus on colistin resistance mediated by only *mcr*. Certain mechanisms of resistance to polymyxins have been described, with the best-known forms involving intrinsic, mutational, and adaptive mechanisms [90]. Because of this heterogeneity, more than one of these mechanisms can possibly be found in a single microorganism; and since certain bacteria can develop antibiotic insensitivity by a process called acquired resistance, while others are naturally resistant to those drugs [91], these additional forms of resistance can explain why *mcr* was not found in all the isolates of *E. coli* that exhibited a resistance to colistin identified by solely phenotypic methods.

## 4. Materials and Methods

### 4.1. Search Strategies 

The systematic review of the literature and the meta-analysis were carried out according to the recommendations of Sagoo, et al. (2009) and as described in Preferred Reporting Items for Systematic Reviews and Meta-Analyses. The following search strategy was applied: in the PubMed database: through the use of the Boolean operator AND with the terms “*Escherichia coli*” OR “*E. coli*” AND “colistin” OR “polymyxins” introduced in the advanced search bar, and the filters activated for the period from 31 December 2014, to March 2021.

### 4.2. Criteria for the Selection of the Studies

The selection of the studies was carried out by two separate reviewers (SS and MV) using the Rayyan QCRI bibliographic manager. Only English-language full-text articles were selected in three phases. The first phase consisted in the removal of all repeated studies. The second phase consisted of the exclusion of articles from the title and abstract review according to the following criteria: (1) studies whose hosts were not pigs, chickens, and/or humans, (2) single-case studies, (3) studies where the bacterial species was different from *E. coli*, (4) studies in which colistin resistance was mediated by a different mechanism from *mcr* genes, (5) Interviews, letters, reviews, or editorials not presenting original data.

The third phase was applied when full texts were read and consisted of a study selection according to the following inclusion criteria: (1) studies where the sampling methods were randomized for all participants, (2) prevalence reports of *mcr*-mediated colistin-resistant *E. coli* (mcrMCRE), where resistance was identified at least by molecular-genetic methods for the presence of *mcr*, (3) clinical studies that included the prevalence of mcrMCRE, (4) animal studies reporting mcrMCRE in which samples were taken from live animals, feces, or carcasses before the processing of meat products. Studies with only phenotypic determinations were left out since we did not know if the *mcr* gene or chromosomal mutations were involved.

### 4.3. Database 

The studies included were divided into two categories: (i) Community studies: those involving pigs, chickens, and healthy humans and (ii) Clinical studies: any investigation related to the hospital environment such as clinical, surgical, and ambulatory cases. For every article selected the following items were collected and introduced into a database in Excel: Title; author(s); year of publication; country; total number of samples collected (n); number of *E. coli* isolates; prevalence of colistin-resistant *E. coli*; and prevalence of mcrMCRE, variants of the *mcr* gene, the host animal, the sample origin, the antimicrobial-susceptibility method used (phenotypic method), or the means of *mcr*-gene identification (molecular-genetic methodology).

### 4.4. Statistical and Meta-Analysis Approach

Descriptive statistics were performed to obtain the crude prevalence of the *mcr* gene according to the continent and host studied. The meta-analysis for the estimation of *mcr*-mediated colistin resistance in *E. coli* was conducted by means of the “meta” package of the R software. A separate meta-analysis was performed for each of the chicken, pig, and human hosts in the community and clinical studies in each region. In total, eight meta-analyses (four meta-analyses each for the origin of the samples and for each continent) were conducted based on a random-effects model. For each prevalence reported, the 95% exact binomial-confidence intervals (95% CI) were calculated. Publication bias was also calculated by a funnel-plot evaluation.

## 5. Conclusions

The results presented here demonstrate a wide dispersion and diversity of *mcr* genes in 54 countries on five continents. We also demonstrate that the majority of the *mcr* genes are in the food chain and most probably play a major role in the dissemination of *mcr* to isolates from humans.

In response to the rapid spread of *mcr* among different hosts, a regular surveillance for colistin resistance is needed to support the practice of evidence-based medicine and a one-health approach.

This study supports the thesis that, since within a common ecosystem microorganisms can affect humans and animals with the same pathology, to contain those contaminants effectively the adoption of an approach that unites animal, human, and environmental health to prevent zoonosis outbreaks and food-safety problems is vitally necessary. Accordingly, an understanding of the epidemiology of colistin-resistant *E. coli* will both facilitate the possibility of formulating prevention protocols and serve to promote comprehensive surveillance worldwide.

## Figures and Tables

**Table 1:** Crude and Estimated Prevalence of *mcr* in *E. coli*.

			Crude Prevalence		Estimated Prevalence	
Data Categories		(n) = *mcr*-*E.coli*/ *E. coli*	%	95% IC	%	95% IC
Host						
	Healthy Humans *	(789/23585)	3.35	3.05–3.65	7.4	3.9–13.6
	Pigs *	(7089/80600)	8.8	8.54–9.06	14.9	10.8–20.1
	Chickens *	(7134/68362)	10.44	10.14–10.74	15.8	11.7–20.9
	Clinical	(1020/58033)	1.76	1.47–2.20	4.2	2.4–7.3
Continent						
	Asia	(8381/90707)	9.2	8.95–9.45	11.5	8.9–14.7
	The Americas	(624/8161)	7.6	6.84–8.36	21.7	7.7–46.9
	Africa	(273/2715)	10.06	9.08–12.12	16.7	8.3–30.9
	Europe	(2305/76137)	3.03	2.87–3.19	9.1	5.7–14.1

* Healthy humans, pigs and chickens were considered as non-clinical samples.

## Data Availability

Not applicable.

## Supplementary Materials

The following supporting information can be downloaded at: https://www.mdpi.com/article/10.3390/pathogens11060659/s1, Figures S1–S8: Forest plot showing the prevalence of mcrMCRE. The X-axis represents the prevalence proportion of the bacteria reported in the individual studies, which are shown on the Y-axis, accompanied by the proportion range of the 95% confidence interval, the proportion of the point estimate and the proportion of the weight of each study in the meta-analysis. The gray squares are the graphical representation of the point estimate for each study, and the line through them represents the 95% confidence interval. The blue parallelogram graphically represents the pooled point estimate of the different groups of the data (Figure S1, Asia; Figure S2, Europe; Figure S3, Africa; Figure S4, The Americas; Figure S5, chickens; Figure S6, Pigs; Figure S7, Healthy humans and Figure S8 clinical samples). Tables S1–S4: Studies included in systematic review and meta-analysis for each sample type (Table S1, chickens; Table S2, pigs; Table S3, healthy humans; and Table S4, clinical samples); Table S5: Prevalence of *mcr* genes in different continents distributed in the selected studies according of data categories. References [92,93,94,95,96,97,98,99,100,101,102,103,104,105,106,107,108,109,110,111,112,113,114,115,116,117,118,119,120,121,122,123,124,125,126,127,128,129,130,131,132,133,134,135,136,137,138,139,140,141,142,143,144,145,146,147,148,149,150,151,152,153,154,155,156,157,158,159,160,161,162,163,164,165,166,167,168,169,170,171,172,173,174,175,176,177,178,179,180,181,182,183,184,185,186,187,188,189,190,191,192,193,194,195,196,197,198,199,200,201,202,203,204,205,206,207,208,209,210,211,212,213,214,215,216,217,218,219,220,221,222,223,224,225,226,227,228,229,230,231,232,233,234,235,236,237,238,239,240,241,242,243,244,245,246,247,248,249,250,251,252,253,254,255,256,257,258,259,260,261 are cited in the supplementary materials.
