# Supplementary material for: Worldwide Prevalence of mcr-mediated Colistin-Resistance Escherichia coli in Isolates of Clinical Samples, Healthy Humans, and Livestock—A Systematic Review and Meta-Analysis
Source: Pathogens. 2022 Jun 8;11(6):659. doi: 10.3390/pathogens11060659 (PMC9230117; doi:10.3390/pathogens11060659)
Supplement: Supplementary file 1 [file pathogens-11-00659-s001.zip › Supplementary tables.pdf]

Supplementary tables

Table S1. Detail of studies of Colistin-resistant *E. coli* mediated by *mcr* genes in chicken's samples included in the meta-analysis.

Author	**Region**	**Microbiological methodology**	**Molecular methodology**	**N**	***E. coli* prevalence**	***mcr* genes**
Joshi, et al. (2019) [92]	AFRICA	BMD/MIC	PCR	324	118	27
Subedi, et al. (2018) [93]	AFRICA	disk diffusion	PCR	50	50	25
Maamar, et al. (2018) [94]	AFRICA	BMD/MIC	PCR	137	48	2
Moawad, et al. (2018) [95]	AFRICA	E-test	PCR	576	63	5
Perreten, et al. (2016) [96]	AFRICA	BMD/MIC	PCR	4934	797	19
Hassen, Abbassi, et al. (2019) [97]	AFRICA	disk diffusion	PCR	333	94	52
Joshi, Thummeepak, et al. (2019) [98]	AFRICA	disk diffusion	PCR	180	60	16
Atterby, et al. (2019) [99]	AFRICA	disk diffusion	PCR	456	129	4
Hassen, et al [100]	AFRICA	BMD/MIC	PCR	286	64	34
Muktan, et al [101]	AFRICA	BMD/MIC	PCR	240	76	21
Dhaouadi, et al [102]	AFRICA	BMD/MIC	PCR	100	50	7
Bista, et al [103]	AFRICA	BMD/MIC	PCR	270	144	18
Ngbede, et al [104]	AFRICA	BMD/MIC	PCR	435	250	17

Büdel, et al [105]	AFRICA	BMD/MIC	PCR	144	69	16
Dominguez, et al. (2018) [106]	AMERICA	disk diffusion	PCR	129	41	31
Dominguez, et al. (2017) [107]	AMERICA	BMD/MIC	PCR	304	304	149
Monte, et al. (2017) [108]	AMERICA	disk diffusion	PCR	41	8	8
Fernandes, et al. (2016) [109]	AMERICA	BMD/MIC	PCR	343	343	10
Vounba, et al. (2019) [110]	AMERICA	BMD/MIC	PCR	327	327	11
Yamamoto, et al. (2019) [111]	AMERICA	E-test	PCR	66	31	15
Saidenberg, et al [112]	AMERICA	BMD/MIC	PCR	64	64	2
Coppola, et al [113]	AMERICA	BMD/MIC	PCR	200	200	1
Eltai, et al. (2018) [114]	ASIA	E-test	PCR	172	90	14
Wu, et al. (2018) [115]	ASIA	BMD/MIC	PCR	821	341	44
Wang, Wang, et al. (2018) [116]	ASIA	BMD/MIC	PCR/ sequencing	102	52	22
Hmede, et al. (2018) [117]	ASIA	BMD/MIC	PCR	93	90	88
Ohsaki, et al. (2017) [118]	ASIA	BMD/MIC	PCR	70	70	1
Lima Barbieri, et al. (2017) [119]	ASIA	BMD/MIC	PCR	980	980	12
Yang, et al. (2017) [120]	ASIA	BMD/MIC	PCR	4934	4934	19
Wang, Zhang, Li, et al. (2017) [121]	ASIA	agar dilution/MIC	PCR	245	161	37

Liu BT, Song, et al. (2017) [122]	ASIA	agar dilution/MIC	PCR	78	78	53
Nakayama, et al. (2017) [123]	ASIA	E-test	PCR	30	7	2
Shen, Wang, et al. (2016) [124]	ASIA	BMD/MIC	PCR	1611	1611	104
Lv, Mohsin, et al. (2018) [125]	ASIA	E-test	PCR	100	100	8
Song, Yu, et al. (2019) [126]	ASIA	disk diffusion	PCR	400	130	75
Zhuge, et al. (2019) [127]	ASIA	BMD/MIC	PCR	1718	1360	172
Li, Sun, et al. (2019) [128]	ASIA	BMD/MIC	PCR	1273	962	962
Liu, Liao, et al. (2018) [129]	ASIA	BMD/MIC	PCR	1723	1723	60
Zhang, et al. (2019) [130]	ASIA	agar dilution/MIC	PCR	2199	1766	388
Liu, Song, et al. (2019) [131]	ASIA	agar dilution/MIC	PCR	493	342	102
Yamaguchi, et al. (2018) [132]	ASIA	agar dilution/MIC	PCR	330	261	56
Yassin, et al. (2017) [133]	ASIA	BMD/MIC	PCR	757	624	13
Nishino, et al. (2017) [134]	ASIA	agar dilution/MIC	PCR	87	87	8
Kawanishi, et al. (2016) [135]	ASIA	agar dilution/MIC	PCR	4513	4513	14
Nguyen, et al. (2016) [136]	ASIA	E-test	PCR	180	180	20
Malhotra Kumar, et al (2016) [137]	ASIA	BMD/MIC	PCR	24	24	2
Oh SS, Song, et al. (2019) [138]	ASIA	BMD/MIC	PCR	93	93	2

Zhang, et al. (2019) [139]	ASIA	BMD/MIC	PCR	207	207	1
Kawahara, et al. (2019) [140]	ASIA	BMD/MIC	PCR	72	72	35
Gao, et al (2019) [141]	ASIA	agar dilution/MIC	PCR	24	24	4
Vinh Trung, et al. (2017) [142]	ASIA	E-test	PCR	714	200	10
Bui, et al. (2018) [143]	ASIA	disk diffusion	PCR	149	124	10
Chen, Zhao, et al. (2017) [144]	ASIA	BMD/MIC	PCR	2330	1709	45
Aklilu, Raman [145]	ASIA	BMD/MIC	PCR	50	23	12
Song, Yu, et al [146]	ASIA	BMD/MIC	PCR	400	130	75
Amin, et al [147]	ASIA	BMD/MIC	PCR	104	104	14
Dutta, et al [148]	ASIA	BMD/MIC	PCR	250	133	33
Xiangkai , et al [149]	ASIA	BMD/MIC	PCR	489	489	61
Zhao, et al [150]	ASIA	BMD/MIC	PCR	722	668	102
Oh, et al [151]	ASIA	BMD/MIC	PCR	34	2	2
Kim, et al [152]	ASIA	BMD/MIC	PCR	818	543	8
Rafique, et al [153]	ASIA	BMD/MIC	PCR	1219	92	13
Ahmed, et al [154]	ASIA	BMD/MIC	PCR	1200	1200	305
Azam, et al [155]	ASIA	BMD/MIC	PCR	75	75	29

Afridi, et al [156]	[ASIA	BMD/MIC	PCR	410	242	1
Liu, et al [157]	ASIA	BMD/MIC	PCR	707	168	41
Javed, et al [158]	ASIA	BMD/MIC	PCR	630	126	90
Yu, et al [159]	ASIA	BMD/MIC	PCR	100	83	53
Sadek, et al [160]	ASIA	BMD/MIC	PCR	345	19	19
Zurfluh, et al. (2017) [161]	EUROPE	BMD/MIC	PCR	1144	3	3
Pietsch, et al. (2018) [162]	EUROPE	BMD/MIC	PCR	164	164	8
El Garch, et al. (2018) [163]	EUROPE	BMD/MIC	PCR	11980	10206	45
Alba, et al. (2018) [164]	EUROPE	BMD/MIC	PCR	604	338	8
Irrgang, et al. (2016) [165]	EUROPE	BMD/MIC	PCR	10609	10609	299
Perrin-Guyomard, et al. (2016) [166]	EUROPE	BMD/MIC	PCR	1450	1450	7
Zajac, et al. (2019) [167]	EUROPE	BMD/MIC	PCR	5878	180	13
Zurfluh, et al. (2017) [168]	EUROPE	BMD/MIC	PCR	320	320	2
Dona, et al. (2017) [169]	EUROPE	BMD/MIC	PCR	545	545	6
Doumith, et al. (2016) [170]	EUROPE	agar dilution/MIC	PCR/ sequencing	2625	1739	2
Maciuca, et al. (2019) [171]	EUROPE	disk diffusion	PCR	107	107	11
Adiguzel, et al [172]	EUROPE	BMD/MIC	PCR	225	225	1

Majewski, et al [173]	EUROPE	BMD/MIC	PCR	158	25	25
Savin, et al [174]	EUROPE	BMD/MIC	PCR	82	82	20
Mesa-Varona, et al [175]	EUROPE	BMD/MIC	PCR	407	407	23
Pesciaroli, et al [176]	EUROPE	BMD/MIC	PCR	825	15	12

BMD: Broth Micro-dilution; MIC: Minimum Inhibitory Concentration; PCR: Polymerase Chain Reaction; CR E. coli: Colistin-Resistant E. coli; *Studies including other hosts but also healthy humans. The table shows the data only for isolates from humans.

Table S2. Detail of studies of colistin-resistant *E. coli* mediated by *mcr* genes in pigs included in the meta-analysis.

Author	Region	Microbiological methodology	Molecular methodology	N	*E. coli* prevalence	*mcr* genes
Atterby, et al (b). (2019)*	Africa	disc diffusion	PCR	456	129	4
Ngbede, et al	Africa	BMD/MIC	PCR	220	65	10
Kieffer, et al. (2018) [177]	America	BMD/MIC	PCR	126	126	1
Yamamoto, et al (b). (2019)*	America	E-test	PCR	66	31	16

Meinersmann, et al (b). (2017)* [178]	America	Vitek-2	PCR	801	801	2
Delgado Blas, et al (b). (2016)* [179]	America	BMD/MIC	PCR / sequencing SANGER	49	49	1
Coppola, et al	America	BMD/MIC	PCR	153	153	8
Wang, et al. (2019) [180]	Asia	agar dilution/MIC	PCR	30	25	25
Dandachi, et al. (2019) [181]	Asia	disc diffusion	PCR	243	105	27
Li, et al. (2018) [182]	Asia	agar dilution/MIC	PCR	417	417	61
Tong, et al. (2018) [183]	Asia	BMD/MIC	PCR	600	457	152
Li, Liu, et al. (2018) [184]	Asia	BMD/MIC	PCR	306	306	78
Strom, et al. (2018) [185]	Asia	BMD/MIC	PCR	261	110	22
Li , Xie, et al. (2017) [186]	Asia	agar dilution/MIC	PCR	97	97	35
Kong, Lei, et al. (2017) [187]	Asia	agar dilution/MIC	PCR	105	105	16
Wang, Li Z,?Lin, et al. (2016) [188]	Asia	BMD/MIC	PCR	1026	1026	302
Kusumoto, Ogura, et al. (2016) [24]	Asia	agar dilution/MIC	PCR	967	684	90
Peng, et al. (2019) [189]	Asia	BMD/MIC	PCR	538	538	8
Do, Park, et al. (2019) [190]	Asia	BMD/MIC	PCR	364	364	9
Shafiq, et al. (2019) [191]	Asia	BMD/MIC	PCR	432	275	197
Fukuda, et al. (2018) [192]	Asia	agar dilution/MIC	PCR	676	676	81

Lai, et al (b). (2018)* [193]	Asia	BMD/MIC	PCR	702	140	12
Liu, Liao, et al (b). (2018)*	Asia	BMD/MIC	PCR	1723	1723	450
Zhang, et al (b). (2019)*	Asia	agar dilution/MIC	PCR	2199	1766	303
Liu, Song, et al (b). (2019)*	Asia	agar dilution/MIC	PCR	493	342	38
Belaynehe, Shin, et al. (2018)* [194]	Asia	BMD/MIC	PCR	636	636	5
Yamaguchi, et al (b). (2018)*	Asia	agar dilution/MIC	PCR	330	261	3
Yassin, et al (b). (2017)*	Asia	BMD/MIC	PCR	757	624	1
Nishino, et al (b). (2017)*	Asia	agar dilution/MIC	PCR	87	87	1
Kawanishi, et al (b). (2016)*	Asia	agar dilution/MIC	PCR	4513	4513	20
Nguyen, et al (b). (2016)*	Asia	E-test	PCR	180	180	17
Malhotra Kumar, et al (b). (2016)*	Asia	BMD/MIC	PCR	24	24	7
Oh SS, Song, et al (b). (2019)*	Asia	BMD/MIC	PCR	93	93	4
Zhang, et al (b). (2019)*	Asia	BMD/MIC	PCR	207	207	1
Kawahara, et al (b). (2019)*	Asia	BMD/MIC	PCR	72	72	34
Yanzheng, et al (b). (2019)*	Asia	agar dilution/MIC	PCR	24	24	12
Chen, Zhao, et al (b). (2017)*	Asia	BMD/MIC	PCR	2330	1709	2
Duggett, et al. (2018) [195]	Europe	BMD/MIC	PCR	349	349	224

Garcia, et al. (2018) [196]	Europe	MicroScan	PCR	186	186	140
Randall, et al. (2018) [197]	Europe	BMD/MIC	PCR/ sequencing	275	275	199
Delannoy, et al. (2017) [198]	Europe	BMD/MIC	PCR	99	99	33
Kieffer, et al. (2017) [199]	Europe	disc diffusion	PCR	100	90	90
Carattoli, et al. (2017) [200]	Europe	agar dilution/MIC	PCR	125	125	46
Hille, et al. (2018) [201]	Europe	BMD/MIC	PCR	127	89	11
Curcio, et al. (2017) [202]	Europe	disc diffusion	PCR	51	51	37
Roschanski, et al. (2017) [203]	Europe	BMD/MIC	PCR	436	43	43
Duggett, Sayers, et al. (2017) [204]	Europe	agar dilution/MIC	PCR	1109	590	2
El Garch, Sauget, et al. (2017) [205]	Europe	disc diffusion	PCR	6274	6274	218
Bai, Hurley, et al. (2016) [206]	Europe	disc diffusion	PCR	1003	93	10
Xavier, Lammens, et al. (2016) [207]	Europe	BMD/MIC	PCR	53	53	11
Xavier, Lammens, Butaye, et al. (2016) [208]	Europe	BMD/MIC	PCR	53	53	7
Quesada, et al. (2016) [209]	Europe	BMD/MIC	PCR	439	439	2
Chabou, et al. (2019) [210]	Europe	SensititreTM	PCR	120	25	25
Garc a Menino, et al. (2019) [211]	Europe	disc diffusion	PCR	499	35	35
Fournier, et al. (2019) [212]	Europe	disc diffusion	PCR	102	62	9

Kieffer, Nordmann, et al. (2019) [213]	Europe	BMD/MIC	PCR	147	147	23
Magistrali, et al. (2018) [214]	Europe	BMD/MIC	PCR	74	74	30
Clemente, et al. (2019) [215]	Europe	agar dilution/MIC	PCR	824	824	42
El Garch, et al (b). (2018)*	Europe	BMD/MIC	PCR	11980	10206	25
Alba, et al (b). (2018)*	Europe	BMD/MIC	PCR	604	338	1
Irrgang, et al (b). (2016)*	Europe	BMD/MIC	PCR	10609	10609	25
Perrin Guyomard, et al (b). (2016)*	Europe	BMD/MIC	PCR	1450	1450	2
Zajac, et al (b). (2019)*	Europe	BMD/MIC	PCR	5878	180	1
Hallenberg, et al (b). (2019) *[216]	Europe	BMD/MIC	PCR	261	261	52

BMD: Broth Micro-dilution; MIC: Minimum Inhibitory Concentration; PCR: Polymerase Chain Reaction; CR *E. coli*: Colistin-Resistant *E. coli*; *Studies including other hosts but also swine. The table shows the data only for isolates from pigs.

Table S3. Detail of studies of Colistin-resistant *E. coli* mediated by *mcr* genes in healthy humans included in the meta-analysis.

Author	Region	Microbiological methodology	Molecular methodology	N	*E. coli* prevalence	*mcr* genes
**Budel, et al. (2019) [217]**	Africa	BMD/MIC	PCR	59	20	10
**Atterby, et al. (a) (2019)***	Africa	disc diffusion	PCR	456	129	1
**Ngbede, et al**	Africa	BMD/MIC	PCR	60	60	8
**Kamweli Aworh, et al [218]**	Africa	BMD/MIC	PCR	110	47	3
**Giani, et al. (2018) [219]**	America	BMD/MIC	PCR	337	170	129
**Delgado Blas, et al. (a) (2016)*** (88)	America	BMD/MIC	PCR / SANGER sequencing	49	49	1
**Berglund, et al. (2018) [220]**	Asia	BMD/MIC	PCR	1000	706	25
**Yamamoto, Kawahara, et al. (2018) [221]**	Asia	BMD/MIC	PCR	98	83	68
**Bi, Berglund, et al. (2017) [222]**	Asia	E-test	PCR	1000	411	20
**Kawahara, et al. (2019) [223]**	Asia	disc diffusion	PCR	612	451	31
**Lai, et al. (a) (2018)***	Asia	BMD/MIC	PCR	702	140	12
**Trung, et al. (a) (2017)***	Asia	E-test	PCR	714	200	2
**Bui, et al. (a) (2018)***	Asia	disc diffusion	PCR	149	124	1
**Chen, Zhao, et al. (a) (2017)***	Asia	BMD/MIC	PCR	2330	1709	3

Johura, et al [224]	Asia	BMD/MIC	PCR	20	20	3
Yamaguchi, et al [225]	Asia	BMD/MIC	PCR	98	57	57
Fukuda, et al [226]	Asia	BMD/MIC	PCR	517	517	1
Del Bianco, et al. (2018) [227]	Europe	Vitek-2	PCR	19053	12441	70
Dona, Bernasconi, et al. (2017)	Europe	SensititreTM	PCR	26	26	3
Leangapichart, et al. (2016) [228]	Europe	E-test	PCR	23	10	10
Vading, et al. (2016) [229]	Europe	Vitek-2	PCR	376	65	1
Hasman, Hammerum, et al. (2015) [230]	Europe	BMD/MIC	PCR	920	920	57
Zogg, et al. (2016) [231]	Europe	BMD/MIC	PCR	80	40	2
Stoesser, et al. (a) (2016) *[232]	Europe	BMD/MIC	PCR	1100	1100	1
Zurfluh, et al. (a) (2017)*	Europe	BMD/MIC	PCR	320	320	3
Dona, et al. (a) (2017)*	Europe	BMD/MIC	PCR	545	545	3
Doumith, et al. (a) (2016)*	Europe	agar dilution/MIC	PCR / SANGER sequencing	2625	1739	13

BMD: Broth Micro-dilution; MIC: Minimum Inhibitory Concentration; PCR: Polymerase Chain Reaction; CR *E. coli*: Colistin-Resistant *E. coli*; *Studies including other hosts but also healthy humans. The table shows the data only for isolates from humans.

Table S4. Detail of studies of Colistin-resistant *E. coli* mediated by *mcr* genes in clinical samples included in the meta-analysis.

Author	Region	Microbiological methodology	Molecular methodology	N	*E. coli* prevalence	*mcr* genes
Poirel, et al. (2018) [233]	AFRICA	BMD/MIC	PCR	50	30	1
Coetzee, et al. (2016) [234]	AFRICA	BMD/MIC	PCR	523	78	19
Muktan, et al [235]	AFRICA	BMD/MIC	PCR	705	56	11
El-Mokhtar, et al	AFRICA	BMD/MIC	PCR	2340	140	21
Saavedra, et al. (2017) [236]	AMERICA	BMD/MIC	PCR	5887	513	8
Walkty, et al. (2016) [237]	AMERICA	BMD/MIC	PCR	5571	5571	2
Faccone, et al [238]	AMERICA	BMD/MIC	PCR	192	192	192
Rocha, et al [239]	AMERICA	BMD/MIC	PCR	100	22	2
Li, Ke, et al. (2018) [240]	ASIA	BMD/MIC	PCR	123	123	6

Feng, Shen, et al. (2018) [241]	ASIA	BMD/MIC	PCR	349	349	88
Lai, Lin, et al. (2018)	ASIA	BMD/MIC	PCR	1359	686	6
Yoon, et al. (2018) [242]	ASIA	BMD/MIC	PCR	9396	2659	2
Lu, Wang, Dong, et al. (2018) [243]	ASIA	BMD/MIC	PCR	3434	3434	12
Shen, Wu, et al. (2018) [244]	ASIA	BMD/MIC	PCR / sequencing	80	80	63
Eiamphungporn, et al. (2018) [245]	ASIA	BMD/MIC	PCR / sequencing	317	37	11
Zhong, Phan, et al. (2018) [246]	ASIA	BMD/MIC	PCR / sequencing	8022	173	70
Manohar, et al. (2017) [247]	ASIA	BMD/MIC	PCR	89	43	11
Wang, Sun, et al. (2017) [248]	ASIA	BMD/MIC	PCR	200	200	10
Wang, Tian, et al. (2017) [249]	ASIA	BMD/MIC	PCR / sequencing	17498	9454	120
Quan, et al. (2017) [250]	ASIA	BMD/MIC	PCR / sequencing	2066	1495	20
Kim, et al. (2017) [251]	ASIA	BMD/MIC	PCR	1193	1193	1
Yu, et al. (2016) [252]	ASIA	BMD/MIC	PCR	264	36	3
Zhong, et al. (2019) [253]	ASIA	BMD/MIC	PCR	144	144	3
Lee, et al. (2019) [254]	ASIA	BMD/MIC	PCR	1184	398	1
La, Lee, et al. (2019) [255]	ASIA	BMD/MIC	PCR	201	17	12
Hsueh, et al [256]	ASIA	BMD/MIC	PCR	291	291	2

Velasco, et al [257]	ASIA	BMD/MIC	PCR	123	2	2
Huang, et al [258]	ASIA	BMD/MIC	PCR	1868	376	14
Javed, et al	ASIA	BMD/MIC	PCR	38500	2219	3
Jiang, et al [259]	ASIA	BMD/MIC	PCR	6401	3598	17
Palani, et al [260]	ASIA	BMD/MIC	PCR	65	3	3
Lalaoui, et al. (2019) [261]	EUROPA	BMD/MIC	PCR	217	4	4
Bourrel, et al. (2019) [262]	EUROPA	BMD/MIC	PCR	1217	165	7
Prim, et al. (2017) [263]	EUROPA	BMD/MIC	PCR	13579	76	15
Juhasz, et al. (2017) [264]	EUROPA	BMD/MIC	PCR	504	86	1
Huang, et al. (2017) [265]	EUROPA	BMD/MIC	PCR	1991	1276	1
Prim, Rivera, et al. (2016) [266]	EUROPA	BMD/MIC	PCR	10011	10011	7
Mariani, Corbella. (2019) [267]	EUROPA	BMD/MIC	PCR	1557	1557	14
Nabti, et al. (2019) [268]	EUROPA	BMD/MIC	PCR	237	237	1
Lellouche, et al (2019) [269]	EUROPA	BMD/MIC	PCR	364	44	40
Mariani, et al [270]	EUROPA	BMD/MIC	PCR	1557	1557	11
Janssen, et al [271]	EUROPA	BMD/MIC	PCR	428	10	10
Wise, et al (2018) [272]	MUNDIAL	BMD/MIC	PCR	44407	64	27

**Ellem, et al. (2017) [273]**	OCEANIA	BMD/MIC	PCR	4555	18	2
**Lalaoui, et al. (2019)**	EUROPA	disk diffusion	PCR	802	217	5
**Zafer, et al. (2019) [274]**	AFRICA	agar dilution/MIC	PCR	450	200	1
**Cao, et al. (2018) [275]**	ASIA	agar dilution/MIC	PCR / hybridization	1112	1112	6
**Farzana, et al. (2019) [276]**	ASIA	agar dilution/MIC	PCR	700	700	1
**Mohsin, et al. (2018) [277]**	ASIA	E-test	PCR	22	2	1
**San, et al. (2019) [278]**	EUROPA	E-test	PCR	1555	442	1
**Henig, et al. (2019) [279]**	AMERICA	SensititreTM / Vitek2	PCR	15894	45	36
**Chan, et al. (2018) [280]**	ASIA	Vitek-2	PCR	672	69	14
**Li, Sun, et al. (2018) [281]**	ASIA	Vitek-2	PCR	7249	229	1
**Luo, et al. (2017) [282]**	ASIA	Vitek-2	PCR / sequencing	1270	1270	21
**He, Xu, et al. (2017) [283]**	ASIA	Vitek-2	PCR / sequencing	700	700	4
**Nijhuis, et al. (2016) [284]**	EUROPA	Vitek-2	PCR	45	18	2
**Principe, et al. (2018) [285]**	EUROPA	Vitek-2	PCR	3902	3902	10
**Newton Foot, et al. (2017) [286]**	AFRICA	Vitek-2 / E-test	PCR	21	14	10

BMD: Broth Micro-dilution; MIC: Minimum Inhibitory Concentration; PCR: Polymerase Chain Reaction; CR *E. coli*: Colistin-Resistant *E. coli*; *Studies including other hosts but also healthy humans. The table shows the data only for isolates from humans.

Table S5. Prevalences of *mcr* genes in different continents distributed in the selected studies according of data categories

	Asia		The Americas		Africa		Europe	
Data Categories	% *mcr* Prevalence (n)	IC 95%	% *mcr* Prevalence (n)	IC 95%	% *mcr* Prevalence (n)	IC 95%	% *mcr* Prevalence (n)	IC 95%
**Comunnity studies**	**14,69 (11375/77459)**	**17,56-18,44**	**21,16 (418/1975)**	**18,79-23,53**	**9,86 (237/2403)**	**9,30-12,12**	**3,29 (2971/90303)**	**5,42-5,82**
Healthy humans	8,3(481/5824)	5,0-7,40	59,8 (131/219)	51,37-68,43	9,0 (22/245)	3,93-12,67	0,9 (155/17222)	0,80-1,20
pigs	12,7 (5054/39940)	18,71-20,29	7,8 (44/561)	4,88-10,72	5,2 (26/499)	5,34-10,46	5,0 (1965/39595)	9,81-10,59
chickens	18,4(5840/31695)	17,64-18,76	20,3 (243/1195)	17,30-23,30	11,4 (189/1659)	10,21-13,79	2,5 (851/33486)	2,38-2,82
**Clinical studies**	**1,54 (735/47611)**	**1,39-1,69**	**3,59 (436/12128)**	**6,96-8,20**	**7,58 (53/699)**	**0-1,22**	**0,52 (144/27600)**	**1,36-1,74**
Blood	1,0 (116/11894)	0,76-1,24	3,4 (194/5763)	2,79-4,01	-	-	0,3 (53/17123)	0,19-0,41
feces	2,2 (416/18724)	1,92-2,48	-	-	3,3 (1/30)	0-11,70	5,7 (19/336)	2,44-8,96
urine	1,8 (132/7412)	1,40-2,20	4 (232/5830)	3,34-4,66	9,1 (30/329)	5,02-13,18	0,1 (7/10011)	0,02-0,18
body fluids	0,02 (2/8510)	0,0-0,06	-	-	6,5 (22/340)	3,06-9,94	100 (10/10)	100-100
respiratory	78,8 (63/80)	67,03-90,57	9,1 (2/22)	1-24,90	-	-	-	-
Others Samples	0,6 (6/991)	0,0-1,23	1,6(8/513)	0,17-3,03	-	-	45,8 (55/120)	34,08-57,52

(n)= Number of *E. coli* isolates positive for *mcr* genes based on molecular methods / number of total of isolates of *E. col*
