# Supplementary figures and images for: Worldwide Prevalence of mcr-mediated Colistin-Resistance Escherichia coli in Isolates of Clinical Samples, Healthy Humans, and Livestock—A Systematic Review and Meta-Analysis
Source: Pathogens. 2022 Jun 8;11(6):659. doi: 10.3390/pathogens11060659 (PMC9230117; doi:10.3390/pathogens11060659)

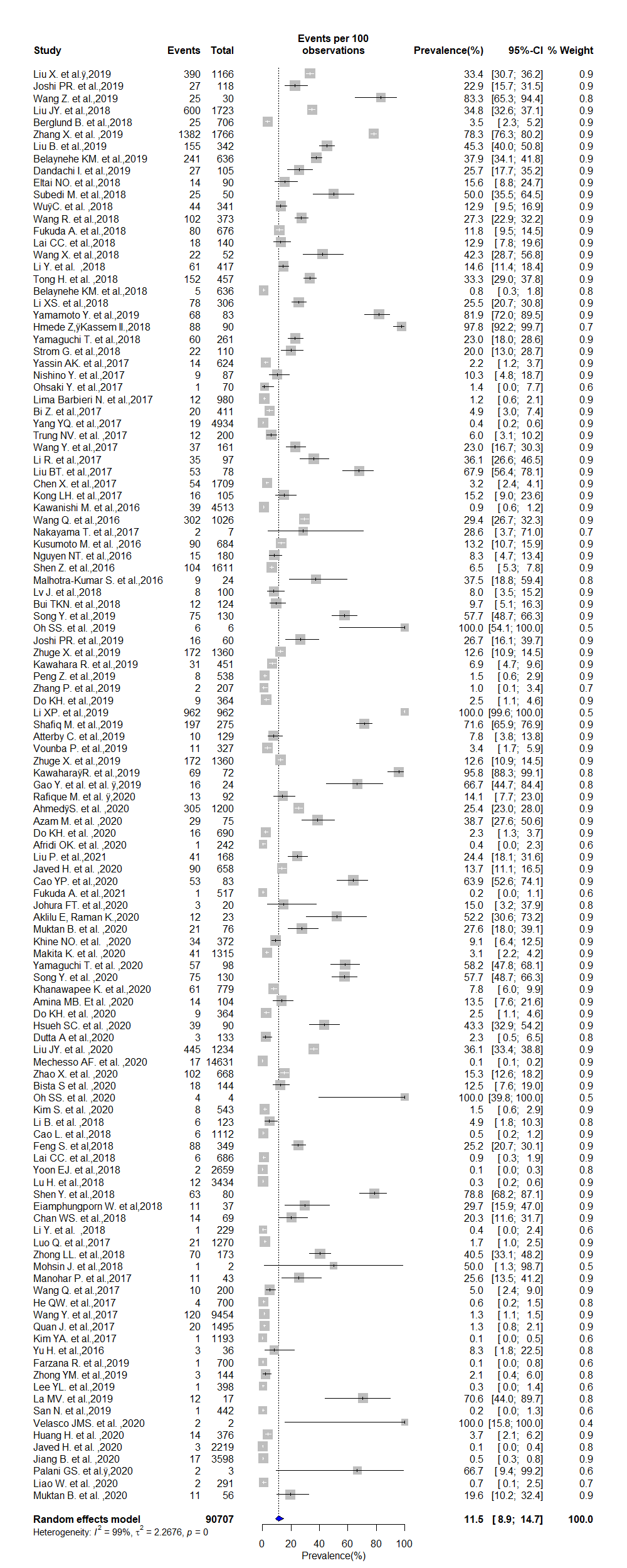

Supplement: Supplementary file 1 [file pathogens-11-00659-s001.zip › Supp.Fig.1.tiff]

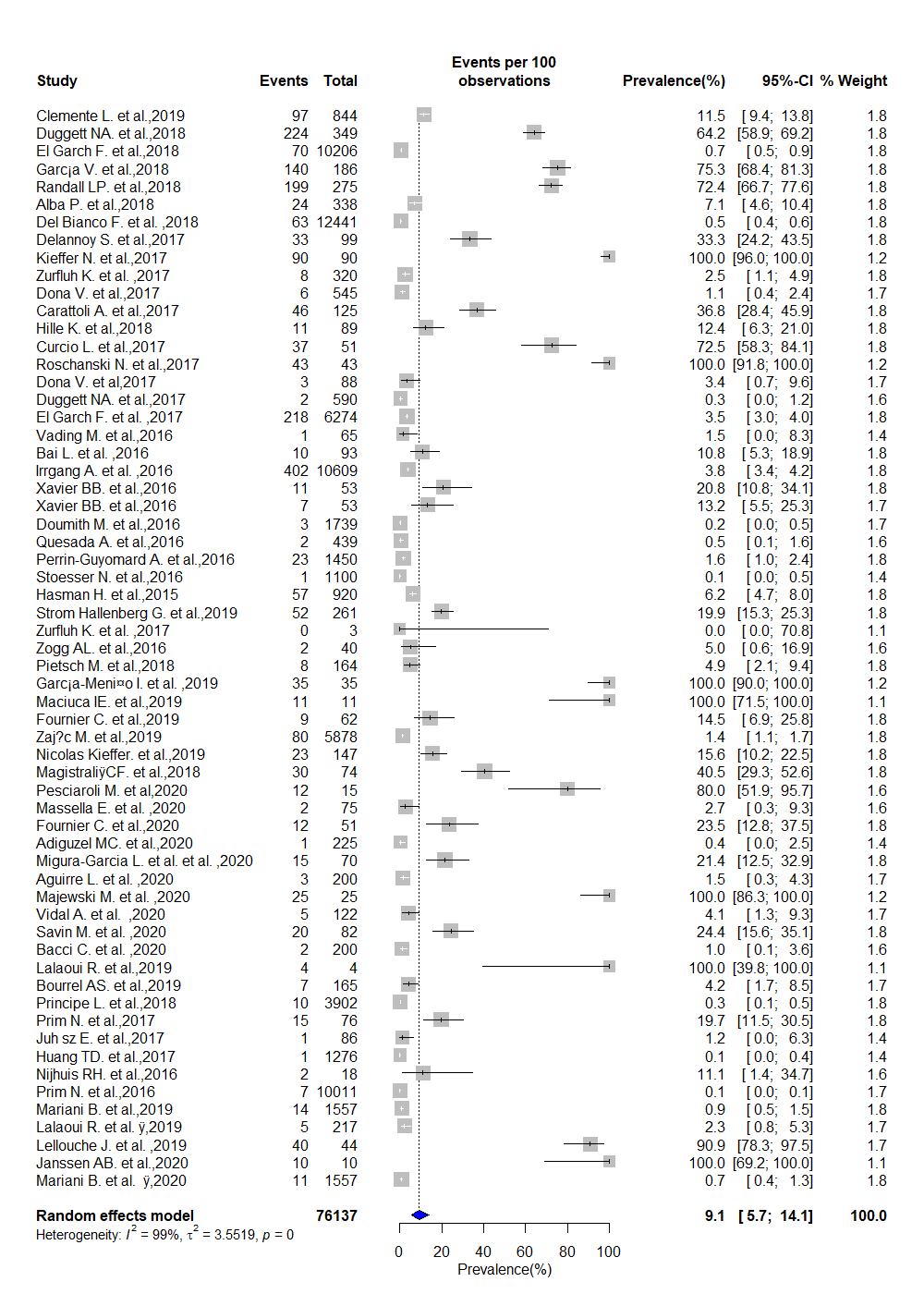

Supplement: Supplementary file 1 [file pathogens-11-00659-s001.zip › Supp.Fig.2.tiff]

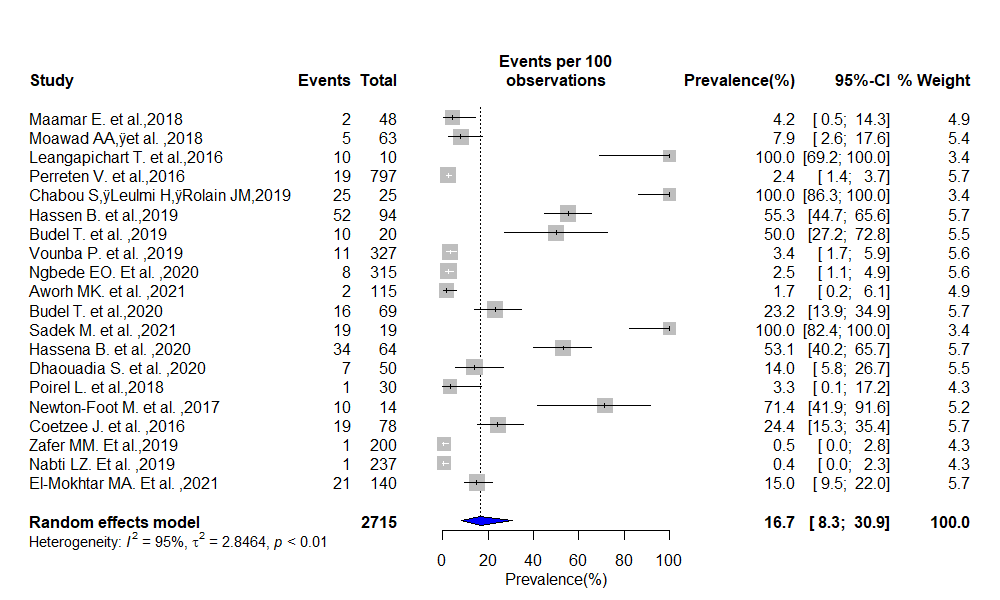

Supplement: Supplementary file 1 [file pathogens-11-00659-s001.zip › Supp.Fig.3.tiff]

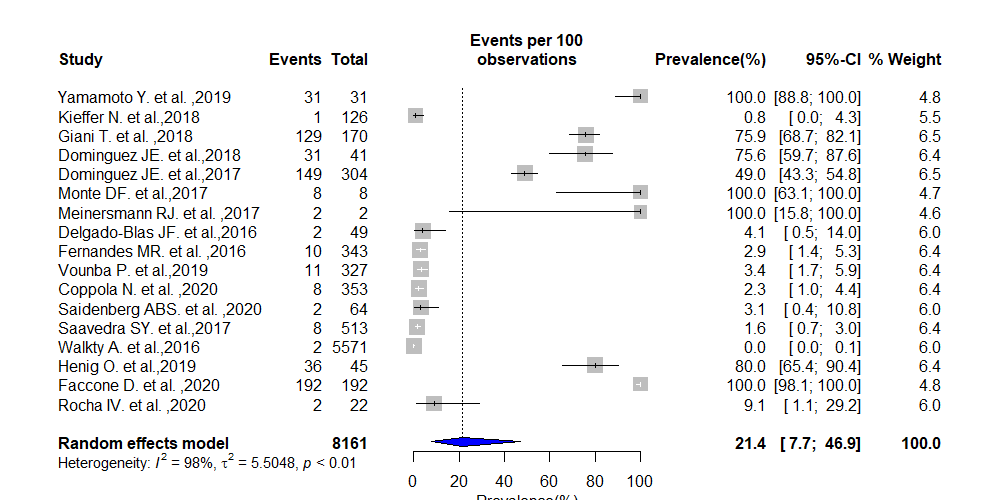

Supplement: Supplementary file 1 [file pathogens-11-00659-s001.zip › Supp.Fig.4.tiff]

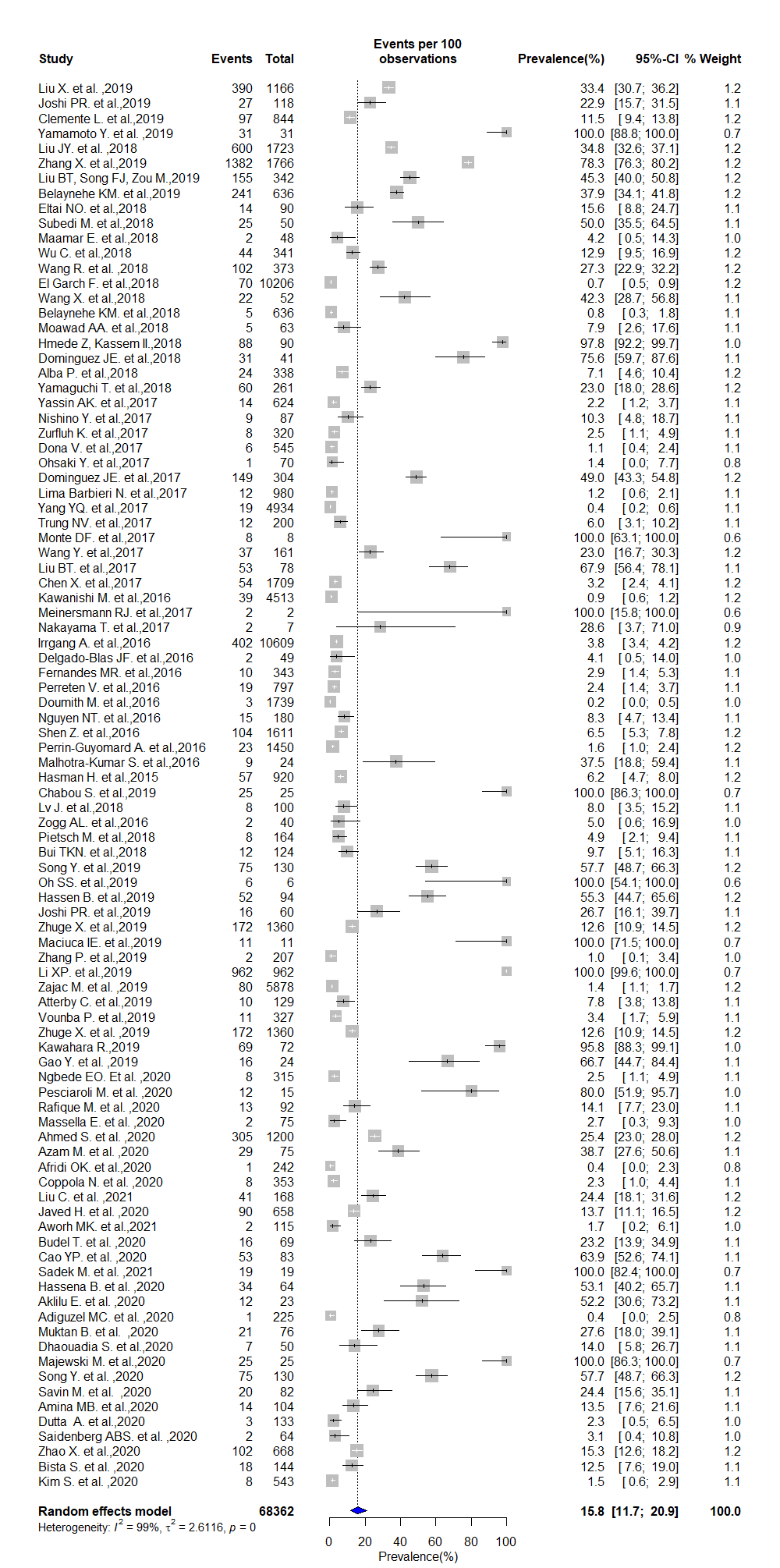

Supplement: Supplementary file 1 [file pathogens-11-00659-s001.zip › Supp.Fig.5.tiff]

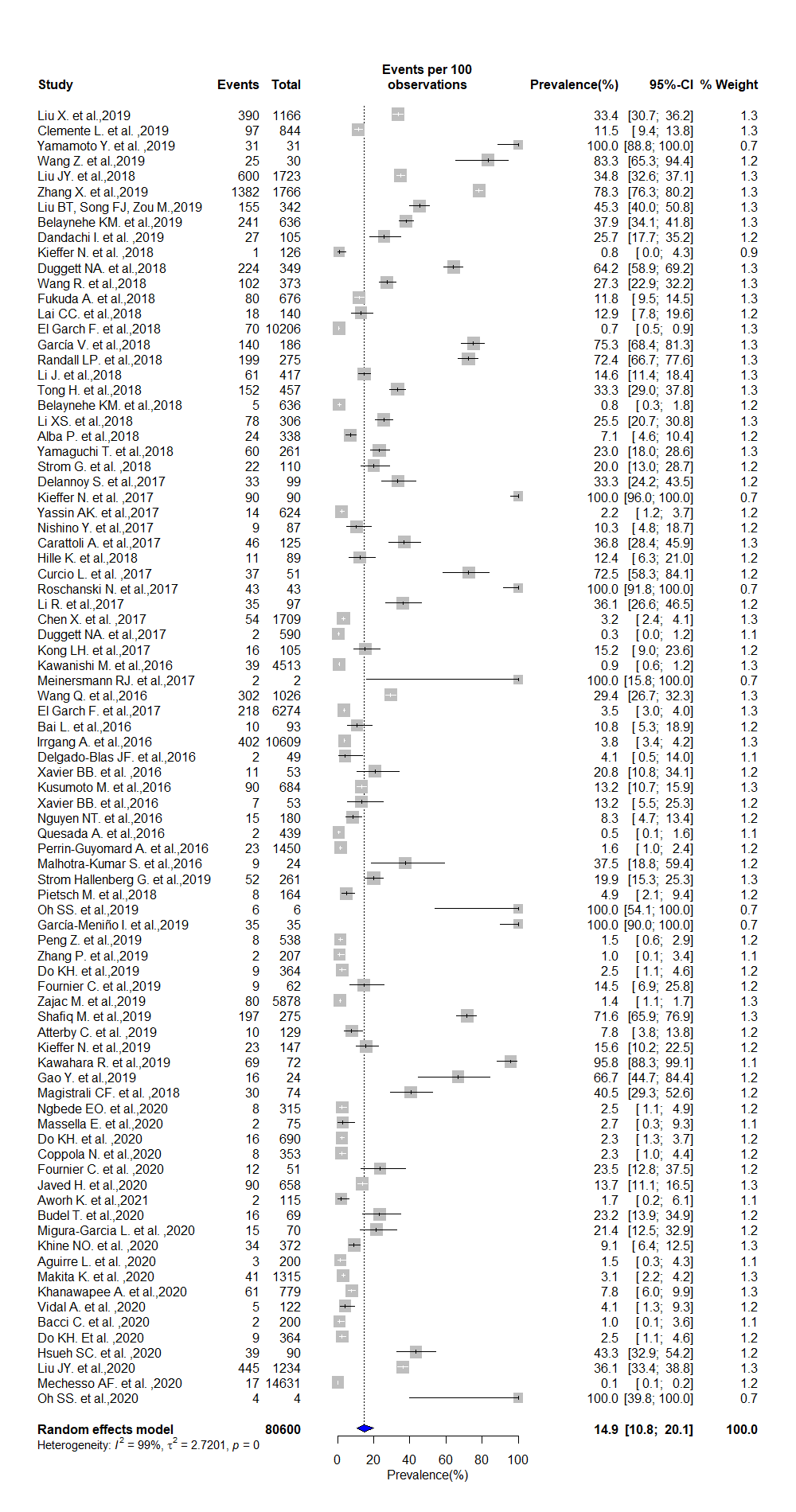

Supplement: Supplementary file 1 [file pathogens-11-00659-s001.zip › Supp.Fig.6.tiff]

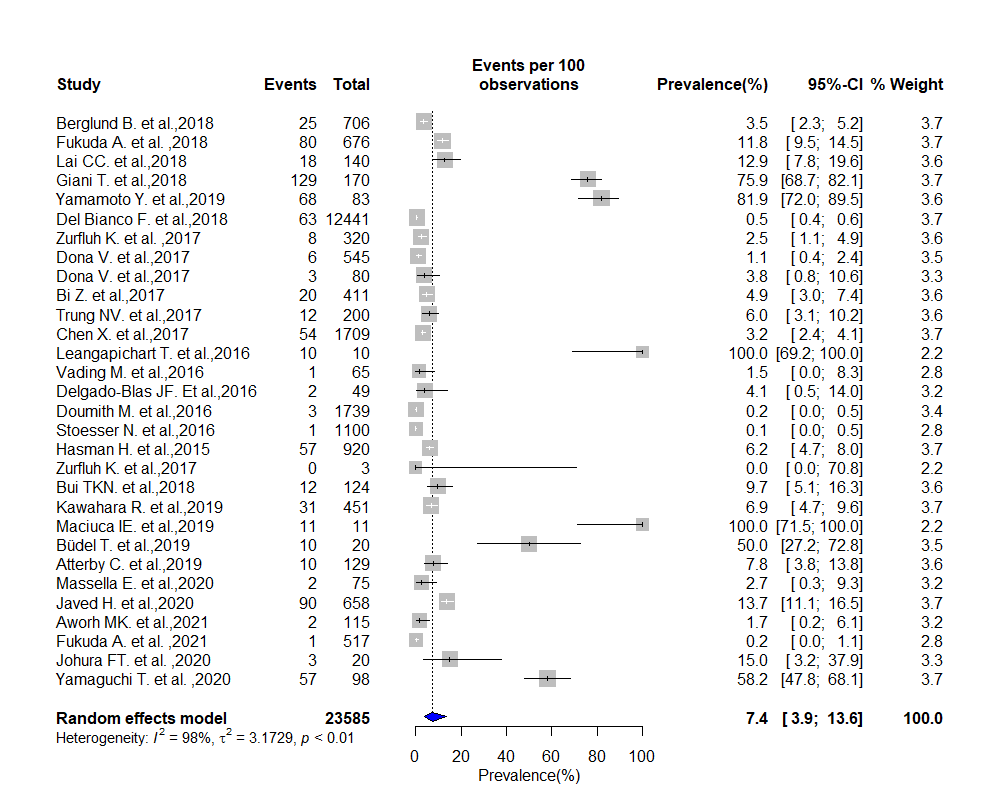

Supplement: Supplementary file 1 [file pathogens-11-00659-s001.zip › Supp.Fig.7.tiff]

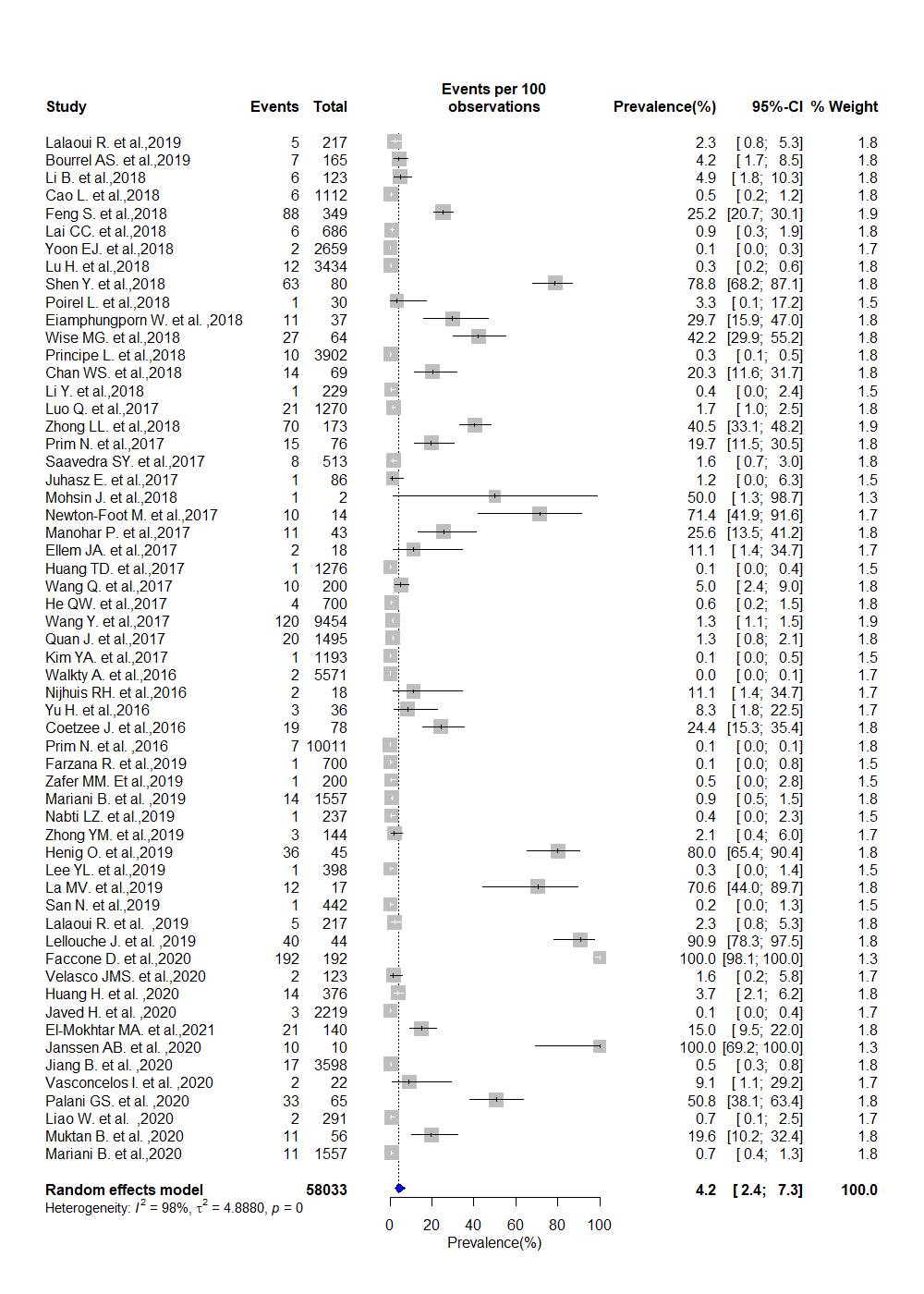

Supplement: Supplementary file 1 [file pathogens-11-00659-s001.zip › Supp.Fig.8.tiff]
